# Smoking and heavy drinking patterns in rural, urban and rural-to-urban migrants: the PERU MIGRANT Study

**DOI:** 10.1186/s12889-017-4080-7

**Published:** 2017-02-03

**Authors:** Alvaro Taype-Rondan, Antonio Bernabe-Ortiz, Germán F. Alvarado, Robert H. Gilman, Liam Smeeth, J. Jaime Miranda

**Affiliations:** 1 CRONICAS Center of Excellence in Chronic Diseases, Universidad Peruana Cayetano Heredia, Armendáriz 497, Miraflores, Lima, 18 Peru; 2 Faculty of Epidemiology and Population Health, London School of Hygiene and Tropical Medicine, London, UK; 3 School of Public Health and Administration, Universidad Peruana Cayetano Heredia, Lima, Peru; 4 Department of International Health, Johns Hopkins Bloomberg School of Public Health, Baltimore, MD USA; 5 School of Medicine, Universidad Peruana Cayetano Heredia, Lima, Peru

**Keywords:** Migration, Alcohol Consumption, Heavy Drinking, Cigarette Consumption

## Abstract

**Background:**

Previous studies have found mixed results about cigarette and alcohol consumption patterns among rural-to-urban migrants. Moreover, there are limited longitudinal data about consumption patterns in this population. As such, this study aimed to compare the smoking and heavy drinking prevalence among rural, urban, and rural-to-urban migrants in Peru, as well as the smoking and heavy drinking incidence in a 5-year follow-up.

**Methods:**

We analyzed the PERU MIGRANT Study data from rural, urban, and rural-to-urban migrant populations in Peru. The baseline study was carried out in 2006–2007 and follow-up was performed five years later. For the baseline data analysis, the prevalence of lifetime smoking, current smokers, and heavy drinking was compared by population group using prevalence ratios (PR) and 95% confidence intervals (95% CI). For the longitudinal analysis, the incidence of smoking and heavy drinking was compared by population group with risk ratios (RR) and 95% CI. Poisson regression with robust variance was used to calculate both PRs and RRs.

**Results:**

We analyzed data from 988 participants: 200 rural dwellers, 589 migrants, and 199 urban dwellers. Compared with migrants, lifetime smoking prevalence was higher in the urban group (PR = 2.29, 95% CI = 1.64–3.20), but lower in the rural group (PR = 0.55, 95% CI = 0.31–0.99). Compared with migrants, the urban group had a higher current smoking prevalence (PR = 2.29, 95% CI = 1.26–4.16), and a higher smoking incidence (RR = 2.75, 95% CI = 1.03–7.34). Current smoking prevalence and smoking incidence showed no significant difference between rural and migrant groups. The prevalence and incidence of heavy drinking was similar across the three population groups.

**Conclusions:**

Our results show a trend in lifetime smoking prevalence (urban > migrant > rural), while smoking incidence was similar between migrant and rural groups, but higher in the urban group. In addition, our results suggest that different definitions of smoking status could lead to different smoking rates and potentially different measures of association. The prevalence and incidence of heavy drinking were similar between the three population groups.

**Electronic supplementary material:**

The online version of this article (doi:10.1186/s12889-017-4080-7) contains supplementary material, which is available to authorized users.

## Background

Cigarette and alcohol consumption are major causes of preventable deaths worldwide. Smoking is the cause of approximately six million deaths per year [1] secondary to the development of different cancers as well as cardiovascular and respiratory diseases [2]. Alcohol consumption, on the other hand, is associated with more than 200 diseases and accidents, causing 5.1% of the global burden of disease [3].

Rural-to-urban migrants are thought to increase their cigarette [4–7] and alcohol [5–9] consumption after migration, not only because they usually migrate from low to high consumption settings, but also because they often suffer from high levels of stress and poor mental health, which are related with greater alcohol and cigarette consumption [10, 11]. Some studies, however, have found that certain rural-to-urban migrants do not consume more alcohol and cigarette products, and even consume less, probably due to a protective effect of rural backgrounds, or economic limitations that prevent substance purchase [12, 13].

Most of the published studies in rural-to-urban migrants have cross-sectional designs, and compare the prevalence of current smoking, alcohol intake, or alcohol intoxication between only two of the three population groups: migrants vs. rural [14] or vs. urban groups [7, 8, 15]. Few cross-sectional studies have contrasted smoking and alcohol consumption in rural-to-urban migrants with their rural groups of origin and their urban counterparts: one study in India [9], two studies in China [6, 16], and one study with data from China, Ghana, India, Mexico, Russia and South Africa [17]. Their results suggest mixed estimates of smoking and alcohol use between rural, migrant and urban groups.

Although cross-sectional data is important to evaluate the association of increased substance consumption between rural-to-urban migrants to their rural and urban counterparts, longitudinal data is necessary to better understand its epidemiology and identify appropriate preventive interventions. To our knowledge, only two longitudinal prospective studies in rural-to-urban migrants have assessed these behavioral risk factors. The first is a study in Tanzania that evaluated smoking rates and weekly alcohol consumption before and one-to-three months after migration [5]. The second is a study in Indonesia that evaluated smoking initiation and changes in smoking quantity among recent (<3 years) migrants [18].

Smoking and alcohol consumption are behaviors whose negative health impacts are closely related to the quantity and frequency of consumption. Yet, before quantifying units of intake, it is also important to address major patterns of consumption across rural, urban, and migrant groups to identify meaningful approaches to prevention.

In addition, epidemiological studies use many different definitions to address substance consumption, which inhibits an adequate comparison. For instance, the concept of “current smoking” has various definitions, and therefore various prevalence rates [19]. Therefore, it is also necessary to compare these definitions and evaluate their appropriateness of use in rural-to-urban migrant studies.

In summary, migrant populations appear to be at increased risk to consume harmful substances such as alcohol and tobacco. To guide regional policy in places of high-density rural-to-urban migration, local studies of substance use are necessary. As such, this study aimed to compare the prevalence of tobacco smoking and heavy drinking among rural, urban, and rural-to-urban migrants in Peru, as well as the incidence of new smoking and new heavy drinking cases at 5-years follow-up.

## Methods

### Study design

This is a secondary data analysis of the PERU MIGRANT Study. The PERU MIGRANT is an ongoing longitudinal study aimed to evaluate cardiovascular risk factors in rural, urban and rural-to-urban migrant population. The methods used have been previously described [20].

### Participants and procedures

Briefly, a random sampling of three different population groups was conducted. 1) Rural population: people born and living in the village of San Jose de Secce, Ayacucho, located at 3,239 m above sea level. 2) Rural-to-urban migrants: people born in rural settings of Ayacucho who have migrated and were living in Lima at enrollment. 3) Urban population: people born and living in Lima, the capital of Peru. Both the urban and migrant populations were taken from Las Pampas de San Juan de Miraflores, a periurban coastal setting located in the south of Lima.

In San Jose de Secce, our rural setting, people usually work as farmers, with hard physical labor and few televisions. In comparison, in our urban setting, people usually work in service industries or factories, and spend their leisure time watching television. In addition, poverty rates and illiteracy are lower in our urban than our rural setting (18 vs 80%, and 2 vs 33%, respectively) [21]. Additional information, including a map of the settings, is available elsewhere [20].

To differentiate rural and urban settings, we relied on population size, according to thresholds proposed by the Department of Agriculture’s Rural–urban Continuum Codes of the United States, that have been extensively used [22, 23]. This institution define rural and urban areas as places with fewer or more than 2,500 inhabitants respectively. Accordingly, in 2006, our urban setting had over 350 thousands and our rural setting less than 1000 inhabitants.

The baseline study was conducted in 2006–2007 after a general census in both study settings. A single-stage random sampling, stratified by sex and age groups, was performed in all three population groups. Trained community health workers administered the questionnaires and collected anthropometric measurements as well as laboratory samples. During 2012–2013, a follow-up visit was carried out, which included a survey and anthropometric measurements.

### Variables definition

#### Cross-sectional outcomes

For the baseline analysis, the outcomes of interest were: lifetime smoking, current smoking, and heavy drinking.

Different definitions of smoking status are currently used in other epidemiological studies. In order to compare these definitions, we calculated prevalence rates for each of our population groups using four of the most popular definitions across the literature (Table 1). For instance, definition 1 is similar to the one used in the Global Adult Tobacco Survey [24] and UK Labour Force Survey [25]. Definition 2 is similar to the one used in the National Survey on Drug Use and Health of United States [19]. Definition 3 is similar to the one used in the CDC’ National Health Interview Survey [26]. Definition 4 is similar to the one used by the New Zealand Ministry of Health [27].

**Table 1:** Prevalence of never, former, and current smokers according to four different definitions

Definitions	Definition 1 Based on having smoked and smoking daily or occasionally		Definition 2 Based on having smoked and havingsmoked in the last month		Definition 3 Based on having smoked at least 100 cigarettes and smoking daily or occasionally		Definition 4 Based on having smoked at least 100 cigarettes and having smoked in the last month	
Never smokers	Experimental smokers or those who have never smoked cigarettes		Experimental smokers or those who have never smoked cigarettes		Those who have not smoked 100 cigarettes in their lifetime		Those who have not smoked 100 cigarettes in their lifetime	
Former smokers	Having smoked, but currently do not smoke daily nor occasionally		Having smoked, but not in the last 30 days		Those who smoked ≥ 100 cigarettes in their lifetime, but currently do not smoke daily nor occasionally		Those who smoked ≥ 100 cigarettes in their lifetime, but currently do not smoke daily nor occasionally	
Current smokers	Those who currently smoke daily or occasionally		Those who have smoked in the last month		Those who smoked ≥100 cigarettes in their lifetime, AND currently smoke daily or occasionally		Those who smoked ≥100 cigarettes in their lifetime, AND have smoked in the last month	
Prevalence rates In our population, per study group	N	% (95% CI)	N	% (95% CI)	N	% (95% CI)	N	% (95% CI)
Prevalence of never smokers								
Urban	61	31.0 (24.5–37.4)	61	31.0 (24.5–37.4)	132	67.3 (60.8–73.9)	132	67.3 (60.8–73.9)
Migrant	231	39.9 (35.9–43.9)	231	39.9 (35.9–43.9)	492	86.2 (83.3–89.0)	492	86.2 (83.3–89.0)
Rural	96	48.0 (41.1–54.9)	96	48.0 (41.1–54.9)	175	93.1 (89.5–96.7)	175	93.1 (89.5–96.7)
Prevalence of former smokers								
Urban	85	43.1 (36.2–50.1)	94	47.7 (40.7–54.7)	29	14.8 (9.8–19.8)	34	17.3 (12.0–22.6)
Migrant	251	43.4 (39.3–47.4)	275	47.5 (43.4–51.6)	39	6.8 (4.8–8.9)	42	7.4 (5.2–9.5)
Rural	41	20.5 (14.9–26.1)	70	35.0 (28.4–41.6)	4	2.1 (0.1–4.2)	5	2.7 (0.4–5.0)
Prevalence of current smokers								
Urban	51	25.9 (19.8–32.0)	42	21.3 (15.6–27.0)	35	17.9 (12.5–23.2)	30	15.3 (10.3–20.3)
Migrant	97	16.8 (13.7—19.8)	73	12.6 (9.9—15.3)	40	7.0 (4.9—9.1)	37	6.5 (4.5—8.5)
Rural	63	31.5 (25.1—37.9)	34	17.0 (11.8—22.2)	9	4.8 (1.7—7.8)	8	4.3 (1.4—7.1)

Although the 100 cigarettes threshold has not been associated with increased clinical risks, it has been extensively used to pragmatically identify lifetime smokers (current or former smokers) and differentiate them from experimental or new-onset smokers, so we decided to use it [28]. In addition, since we are evaluating consumption patterns across two settings, the “occasionally smoking” term could be interpreted differently between people living in these diverse environmental settings. As such, we used the “one-month smoking” threshold to more objectively identify current smokers. Consequently, definition 4, which includes both 100-cigarettes and one-month smoking thresholds, was used to define lifetime smoking and current smoking outcomes for our research questions and statistical models.

Heavy drinking was assessed by the question “In the last year, how often have you consumed 6 or more standard alcohol drinks on one occasion?” Those who answered “monthly,” “weekly,” or “daily or almost daily” were classified as “heavy drinkers.” One standard alcohol drink, as established by the National Institute on Alcohol Abuse and Alcoholism, is defined as a 12 oz (355 mL) glass of beer, a 5 oz (148 mL) cup of wine, or 1.5 oz (44.3 mL) of distilled spirits, [29].

In our settings, people commonly smoke branded cigarettes, and drink either branded beer or artisanal *cañazo* (sugarcane brandy) [30]. *Cañazo* is considered as a distilled spirit, since its alcohol concentration is approximately 40%.

#### Longitudinal outcomes

For the longitudinal analysis, we used two outcomes: new smokers and new heavy drinkers. New smokers were individuals classified as never smokers in the baseline survey who reported having smoked in the last month during the follow-up survey. New heavy drinkers were individuals who denied heavy drinking or did it less than monthly in the baseline, but reported heavy drinking at least monthly at follow-up.

#### Exposure

For both, the cross-sectional and the longitudinal analyses, the exposure of interest was the study group, categorized as rural, urban, and rural-to-urban-migrant groups.

#### Other variables

Other smoking-related variable was daily smoking, defined as participants who answered “I smoke at least a cigarette per day.” to the question “At present, how often do you smoke cigarettes?” Average and median number of daily cigarettes smoked were also estimated among daily smokers.

Demographic variables included in the analyses as potential confounders were: age (<50 or ≥50 years), sex, education level (none or some primary education, complete primary education, and at least some secondary education), possessions weighted assets index, and positive mental health (PMH).

Assets index was based on the number of assets available at the participant’s household, divided in tertiles for each population group (lowest, middle, and highest), and then combined in one single variable. PMH, an expression of a healthy mind, was measured by an adaptation of the General Health Questionnaire (GHQ-12), and treated as a continuous variable, as detailed elsewhere [31].

### Statistical analysis

For the descriptive analysis, means and standard deviations (SD), medians and interquartile ranges (IQR), as well as frequencies and percentages, were utilized. We performed bivariate analyses in order to compare sex, age, education level, assets index, PMH, and daily smoking according to population groups, using Chi-squared or ANOVA tests. We also used the Kruskal-Wallis test to compare the number of daily cigarettes smoked among daily smokers according to population groups.

For cross-sectional analysis, we generated crude and adjusted Poisson regression models with robust variance and estimated prevalence ratios (PR) and 95% confidence intervals (95% CI) in order to assess the associations between exposures (population groups, sex, age, education level, asset index, and PMH) and three dichotomous outcomes: lifetime smoking, current smoking, and heavy drinking. Adjusted models included all exposures mentioned.

For longitudinal analysis, we performed Poisson regression models to report risk ratios (RR) and 95% CI for two outcomes: incidence of new smokers and incidence of new heavy drinkers. For both associations, we generated crude and adjusted models using the same aforementioned exposures and confounders as in cross-sectional models.

We also made post-hoc analyses in the migrant group, which was categorized according to the time since first migration at the baseline survey (<15 years, 15 to 30 years, or >30 years). In each of these categories, prevalence and incidence rates of smoking and of heavy drinking were calculated. Fisher’s exact test was used to evaluate differences in these categories.

### Ethical considerations

Ethical approval for the baseline study was obtained from Institutional Review Boards at Universidad Peruana Cayetano Heredia, in Lima, Peru, and the London School of Hygiene and Tropical Medicine, in London, United Kingdom. The follow-up phase was reviewed and approved by the same Peruvian institution. All enrolled participants gave written informed consent.

## Results

### Population characteristics

We analyzed data from 988 participants: 200 rural, 589 urban-to-rural migrants, and 199 urban residents. Sex was evenly distributed across all three groups. Median ages (IQR) were 47 (37–57), 46 (39–55), and 48 (38–56) years old in the rural, migrant, and urban groups, respectively. The proportion of those having completed at least a year in secondary education was lower in the rural group, intermediate in the migrant group, and higher in the urban group (Table 2).

**Table 2:** Baseline characteristics of the study population, per study group

Variables	Rural (*n* = 200) N (%)	Migrant (*n* = 589) N (%)	Urban (*n* = 199) N (%)	*p*
Women	106 (53.0)	309 (52.5)	107 (53.8)	0.949
Age ≥ 50 years	84 (42.0)	252 (42.8)	89 (44.7)	0.846
Education level				<0.001
None or some primary education	132 (66.0)	183 (31.1)	13 (6.6)	
Complete primary education	29 (14.5)	99 (16.8)	23 (11.6)	
At least some secondary education	39 (19.5)	306 (52.0)	162 (81.8)	
Assets index				<0.001
Lowest	123 (61.5)	242 (41.1)	67 (33.7)	
Middle	14 (7.0)	156 (26.5)	69 (34.7)	
Highest	63 (31.5)	191 (32.4)	63 (31.7)	
Positive mental health score	5.9 ± 1.9	6.5 ± 1.8	6.8 ± 1.8	<0.001
Daily smokers	1 (0.5)	15 (2.6)	17 (8.6)	<0.001

*P* values were calculated using the chi-squared or the ANOVA test

We evaluated four smoking status definitions. The prevalence of never smoking was higher among the rural population than the migrant/urban populations, for all evaluated definitions. The prevalence of current smoking followed a trend (urban > migrant > rural) as per definitions 3 and 4 (which included the 100 cigarettes threshold), was higher in the rural group than in the migrant/urban groups as per definitions 1 and 2. The prevalence of current smoking in definition 1 (defined as having smoked occasionally or daily) was 21.6%, 33.3%, and 85.3% higher than the prevalence of current smokers in definition 2 (defined as having smoked in the last month), among urban, migrant, and rural subjects respectively (Table 1).

The prevalence of daily smoking was higher in the urban group, intermediate in the migrant group, and lower in the rural group. Within daily smokers, the median of cigarettes smoked per day was 1.0 in the rural group, 2.0 in the migrant group, and 2.7 in the urban group (Kruskal-Wallis test *p* = 0.67).

### Cross-sectional and longitudinal models for smoking

In the adjusted model, compared with the migrant group, the prevalence of lifetime smoking was 129% higher (*p* < 0.01) among urban dwellers, and 45% lower (*p* = 0.047) among rural dwellers. In addition, our adjusted model with the migrant group as reference revealed a 129% higher (*p* < 0.01) prevalence of current smoking among the urban group, but showed no significant differences with the rural group (*p* = 0.214) (Table 3).

**Table 3:** Factors associated with lifetime smoking, current smoking, and heavy drinking

Variables	Lifetime smoking		Current smoking		Heavy drinking	
	Crude PR (95% CI)	Adjusted^a^PR (95% CI)	Crude PR (95% CI)	Adjusted^a^ PR (95% CI)	Crude PR (95% CI)	Adjusted^a^ PR (95% CI)
Study group						
Urban	**2.36 (1.77–3.14)**	**2.29 (1.64–3.20)**	**2.36 (1.50–3.72)**	**2.29 (1.26–4.16)**	1.08 (0.63–1.83)	0.91 (0.49–1.68)
Migrant	Ref	Ref	Ref	Ref	Ref	Ref
Rural	**0.50 (0.28–0.88)**	**0.55 (0.31–0.99)**	0.66 (0.31–1.39)	0.60 (0.27–1.33)	1.41 (0.88–2.27)	1.19 (0.71–2.00)
Sex						
Female	Ref	Ref	Ref	Ref	Ref	Ref
Male	**6.04 (3.99–9.16)**	**6.05 (3.78–9.69)**	**6.39 (3.41–11.97)**	**7.08 (3.35–14.95)**	**6.92 (3.81–12.57)**	**6.53 (3.41–12.47)**
Age						
< 50 years	Ref	Ref	Ref	Ref	Ref	Ref
≥ 50 years	1.15 (0.87–1.54)	1.24 (0.91–1.68)	0.83 (0.53–1.29)	1.00 (0.61–1.63)	**0.57 (0.37–0.89)**	**0.55 (0.35–0.88)**
Education level						
None or some primary education	Ref	Ref	Ref	Ref	Ref	Ref
Complete primary education	**2.12 (1.18–3.82)**	1.33 (0.70–2.55)	**2.83 (1.22–6.57)**	1.38 (0.58–3.26)	1.27 (0.66–2.45)	0.74 (0.37–1.48)
At least some secondary education	**3.68 (2.34–5.79)**	1.53 (0.87–2.69)	**3.77 (1.88–7.53)**	0.98 (0.44–2.19)	1.54 (0.95–2.48)	0.77 (0.44–1.33)
Assets index						
Lowest	Ref	Ref	Ref	Ref	Ref	Ref
Middle	**1.83 (1.24–2.70)**	1.26 (0.83–1.90)	1.62 (0.89–2.95)	1.17 (0.60–2.29)	1.09 (0.64–1.86)	1.02 (0.59–1.78)
Highest	**2.14 (1.51–3.05)**	**1.48 (1.02–2.16)**	**2.23 (1.32–3.75)**	1.58 (0.88–2.85)	1.44 (0.91–2.27)	1.16 (0.72–1.87)
Positive mental health (continuous variable)	**1.15 (1.03–1.28)**	0.95 (0.87–1.04)	**1.22 (1.02–1.46)**	1.02 (0.86–1.22)	1.16 (0.99–1.36)	1.06 (0.91–1.25)

^**a**^Adjusted by all the variables listed in the table

Bold numbers indicate significant associations, *p* < 0.05

From the longitudinal adjusted models, among those classified as never smokers during the baseline survey, the risk of smoking in the last month during the 5-year follow up was 175% higher in the urban group than the migrant group (*p* = 0.043), with no significant differences between the migrant and rural groups (*p* = 0–349) (Table 4).

**Table 4:** Risk factors for smoking and heavy drinking incidence

Variables	New smoking			New heavy drinking		
	Incidence	Crude RR (95% CI)	Adjusted^a^ RR (95% CI)	Incidence	Crude RR (95% CI)	Adjusted^a^ RR (95% CI)
Study group						
Urban	15/145 = 10.3%	**2.65 (1.03–6.81)**	**2.75 (1.03–7.34)**	5/155–3.2%	1.48 (0.51–4.25)	1.27 (0.39–4.11)
Migrant	15/475 = 3.2%	Ref	Ref	10/463 = 2.2%	Ref	Ref
Rural	9/158 = 5.7%	1.90 (0.76–4.74)	1.57 (0.61–4.05)	6/153 = 3.9%	1.50 (0.55–4.05)	1.14 (0.40–3.27)
Sex						
Female	9/444 = 2.0%	Ref	Ref	2/446 = 0.4%	Ref	Ref
Male	30/334 = 9.0%	**3.93 (1.66–9.30)**	**4.43 (1.64–11.99)**	19/325 = 5.8%	**12.99 (3.05–55.41)**	**12.25 (3.06–49.03)**
Age						
< 50 years	28/448 = 6.3%	Ref	Ref	14/441 = 3.2%	Ref	Ref
≥ 50 years	11/330 = 3.3%	0.45 (0.18–1.10)	0.53 (0.19–1.45)	7/330 = 2.1%	0.67 (0.27–1.64)	0.75 (0.27–2.03)
Education level						
None or some primary education	6/265 = 2.3%	Ref	Ref	4/261 = 1.5%	Ref	Ref
Complete primary education	7/116 = 6.0%	**4.20 (1.26–14.06)**	2.72 (0.68–10.85)	3/115 = 2.6%	1.77 (0.40–7.79)	0.87 (0.16–4.65)
At least some secondary education	26/396 = 6.6%	2.78 (0.92–8.33)	1.36 (0.33–5.57)	14/393 = 3.6%	2.47 (0.82–7.42)	0.92 (0.25–3.36)
Assets index						
Lowest	10/340 = 2.9%	Ref	Ref	5/336 = 1.5%	Ref	Ref
Middle	12/187 = 6.4%	1.38 (0.50–3.81)	0.97 (0.32–2.97)	7/191 = 3.7%	2.56 (0.82–7.94)	2.29 (0.55–9.62)
Highest	17/251 = 6.8%	1.67 (0.69–4.03)	1.44 (0.56–3.70)	9/244 = 3.7%	2.51 (0.85–7.40)	3.00 (0.78–11.53)
Positive mental health (continuous variable)		1.14 (0.90–1.43)	0.98 (0.76–1.25)		1.11 (0.87–1.40)	0.88 (0.65–1.19)

^a^Adjusted by all the variables listed in the table

Bold numbers indicate significant associations, *p* < 0.05

### Cross-sectional and longitudinal models for heavy drinking

Heavy drinking prevalence was similar among rural and urban groups, when compared to the migrant reference group (Table 3). Likewise, among those who did not report heavy drinking in the baseline survey, the risk of heavy drinking during follow-up was similar among rural and urban groups, when compared to the migrant reference group (Table 4).

### Cross-sectional and longitudinal models for other variables

In the baseline analysis, the prevalence of lifetime smoking, current smoking, and heavy drinking, were higher in men than women (*p* < 0.01). Heavy drinking prevalence was lower in participants older than 50 years old (*p* = 0.013). Higher prevalence of lifetime smoking was noted among those with a higher assets index (*p* = 0.041) (Additional file 1: Table S1 and Table 3). In the longitudinal analysis, the incidence of new smoking and new heavy drinking were higher among men than among women (*p* < 0.01) (Table 4).

## Discussion

### Main results

We found that lifetime smoking prevalence was higher in urban dwellers, intermediate in migrants, and lower in rural dwellers. This indicates that following on a process of internal migration, subjects are more exposed to smoking behaviors than rural dwellers, and experience more smoking. However, smoking incidence was not different between rural and migrant groups. In comparison, prevalence and incidence of heavy drinking were similar between rural, migrant and, urban groups.

### Smoking patterns

When comparing four “current smoking” definitions, it is clear that prevalence found with definitions that asked for “occasionally or daily smoking” tend to be higher than the prevalence found with definitions that asked for “smoking in the last month”. Moreover, these differences seem to be higher among rural dwellers, suggesting that more objective definitions of current smoking are needed, especially in low-consumption settings.

In addition, in definitions 1 and 2, which did not utilize the 100 cigarettes threshold, no trend was observed. Implying, perhaps, that secular changes are occurring, and rural dwellers have recently started smoking; thus, they endorsed smoking in the last month but denied smoked more than 100 cigarettes in their lifetime. Although not statistically significant, this seems to be reinforced by the higher, smoking incidence in the rural group.

Our population has a low smoking prevalence, consistently with previous studies conducted in Peru [32, 33]. Conversely, prevalence of daily smoking in high income countries reaches 13.7% in United States [34] and 25% in Finland [35]. Among daily smokers, the mean of cigarettes per day consumed by our population was under five cigarettes per day, lower than in other Latin American cities as Santiago (Chile), Quito (Ecuador), Bogota (Colombia), and Mexico City [33], and other countries as United States [26] and China [36].

Smoking prevalence and incidence rates were lower in migrants than in the urban population, which suggest that the rural background have a protective effect. Lifetime smoking was significantly higher in the migrant than in the rural group. This smoking pattern is similar to that found with Chinese rural-to-urban migrants [4], where behavior habit adoption has been attributed to acculturation, stress [11] and poor mental health [37]. Conversely, in the adjusted analysis, PMH, a mental health status evaluation, was not associated with smoking prevalence or incidence. However, since our migrant group lived in an urban environment for an average of 32 years, it is possible we are not observing an association of PMH that is given only among more recent migrants. On the other hand, current smoking was not significantly different among rural and migrant groups, possibly due to the low prevalence of current smokers and the small sample size.

Smoking prevalence rates followed a consistent trend in our population (urban > migrant > rural). We found four studies that also compared smoking prevalence rates between the rural-to-urban migrants, urban, and rural groups. A study in Yi migrants (China) found the same trend but only in men, while smoking prevalence among women was higher in the migrant group than in the rural and urban groups [6]. A multi-country study found that migrants had a higher ever smoking prevalence than rural dwellers in Mexico, while no significant difference was found in China, Ghana, India, Russia, and South Africa [17]. These mixed results may be explained by differences in time since migration, smoking patterns, or the acculturation process between population groups included in those studies and ours. In addition, studies in India [9] and China [16] found that male migrants had lower cigarette consumption than rural and urban males. In these last studies, the migrant group was composed of workers, so it is possible that selective migration of people with the best education and predisposition to improve their lifestyle would explain their low consumption, while migration in our study was influenced by political violence lived in Ayacucho [38, 39] which could have reduced this selective migration effect.

From the longitudinal point of view, only two prospective studies evaluating smoking in rural-to-urban migrants were found. A study in Tanzania [5] compared current smoking rates before migration and one to three months after migration, and found a non-significant increase in smoking rates (from 16.2 to 23.5%) only in men, while no women reported smoking in either evaluation. Another study in Indonesia [18] followed-up recent migrants, approximately 65% migrating less than three years ago, and found no significant increase in smoking initiation, but a clear increase in the number of daily cigarettes smoked. These studies suggest a slight increase in smoking rates after migration, but give no information about risk in long-term settled migrants.

While lifetime smoking prevalence was higher in migrants than in the rural population, smoking incidence was not significantly different between those populations. This may suggest that the risk of initiating smoking in migrants could increase during the first years post-migration, and later decrease over time. To evaluate this assumption, we made a post-hoc analysis in our migrant group, and found that prevalence rates of having smoked in the last month were 0.0, 14.9, and 11.3% among those who migrated <15, 15 to 30, and >30 years prior to the baseline assessment (Fisher’s exact *p* = 0.244). Accordingly, incidence rates were 0.0, 3.5, and 1.3% in these sub-groups (Fisher’s exact *p* = 0.334). These findings suggest a higher smoking risk at 15 to 30 years of migration.

### Heavy drinking

The prevalence of heavy drinking in the last year was similar between the urban, migrant, and rural groups. Accordingly, studies that evaluated alcohol intake in Guatemala [14] and alcohol dependence in Canada [40], found similar patterns between rural-to-urban migrant and rural groups. However, data from Tanzania [5] found that weekly alcohol consumption prevalence increased after migration, and studies assessing monthly drinking in Vietnam [7] and alcohol intoxication in China [15] found that rural-to-urban migrants had higher rates than the urban population.

Three previous studies have compared alcohol intake rates between the rural-to-urban migrants, urban, and rural groups. One of them [6] made in Yi migrants (China), found that migrants had similar prevalence rates of current alcohol use than rural and urban dwellers. The other study, also in China [16], surveyed migrants recruited in workplaces and reported that they had higher alcohol intoxication rates than rural and urban dwellers. While the first study resembles our results, the second did not, possibly because their participants were workers, younger (mean age 25 years), and therefore possibly more prone to alcohol drinking, than our migrant group with mean age of 48 years. The third study found similar alcohol use between rural and migrant groups in Ghana, India, Mexico, Russia, and South Africa, while migrants had lower alcohol use than rural dwellers in China [17].

We only found one longitudinal study that evaluated alcohol intake in rural-to-urban migrants in Tanzania, which found that weekly alcohol consumption has a non significant increase after migration [5]. In our longitudinal analysis, incidences of heavy drinking were similar between the three study groups. As for smoking, we verified whether heavy drinking increases after the first years of migration in post-hoc analysis in our migrant group, and found that prevalence rates of heavy drinking were 18.2, 7.1, and 8.6% among those who migrated <15, 15 to 30, and >30 years previous to baseline assessment (Fisher’s exact *p* = 0.270). However, incidence rates were 0.0, 3.9, and 0.8 in these sub-groups (Fisher’s exact *p* = 0.099). These findings, along with the Tanzania study, are consistent with a non-significant increase in heavy drinking in the first years after migration.

Subjects in our rural and urban settings, although living in different socio-environmental contexts, had similar heavy drinking rates. This may reflect similar heavy drinking manners in our urban and rural populations, along with similar alcohol access besides economic differences, probably because low resource individuals in our settings can purchase low-cost artisanal alcoholic drinks [30]. Some studies suggest that recent migrants may present migration-related psychological distress, which was associated to higher alcohol consumption [41, 42]. However, PMH, a mental health status evaluation, was not associated to heavy drinking in late-term migrants.

Classically, smoking has been associated with alcohol intake and with heavy drinking [43, 44]. However, in Peru, alcohol intake prevalence is much higher than smoking prevalence [45], and our results show that increasing smoking rates in migrants are not accompanied by an increase in heavy drinking rates. A possible explanation is that low-resource populations do not have enough money to buy cigarettes, but they can manufacture low-cost artisanal alcohol drinks [30]. Accordingly, asset index was associated with the prevalence of lifetime smoking, but not with the prevalence of heavy drinking.

### Public health relevance

Our results present smoking and heavy drinking patterns in a rural-to-urban internal migration in Peru, which may be similar to other rural-to-urban internal migrations in Peru and other developing countries.

Our results indicate that migrants are at risk to increase their smoking patterns, especially in the first years after migration. Thus, smoking interventions in migrant populations appear more beneficial if oriented to prevent smoking initiation rather than cessation. These observations are not against major tobacco control policies that remain to be sustained as beneficial public health policies at the country-level [46].

Post-hoc analyses show not significant lower smoking rates and higher heavy alcohol drinking rates among those who migrated in the past 15 years. Future studies in recent migrants could identify which would be the best moment for preventive interventions in smoking and alcohol consumption.

It is also important to take into account that Peruvian rural settings could have a higher use of artisanal alcohol distilled drinks with high alcohol concentration [30], which may have a higher concentration of aliphatic alcohol [47], and therefore represent an additional risk of liver damage [48].

### Strengths and limitations

This study has assessed smoking and heavy drinking in well-defined rural, urban, and migrant populations. This allows a better understanding of the influence of rural–urban migration in the consumption patterns, and can be used to improve health interventions targeted towards these migrants.

However, some limitations deserve consideration. First, all the variables studied were self-reported, with the inherent social desirability bias. Nevertheless, previous studies in other countries reinforce use of self-reporting as a reliable method to measure the smoking status [49–51] and alcohol consumption [52, 53] in the general population. Second, there are some confounders that we could not address, such as smoking/drinking status before migration, or reason of migration. Finally, some studies have reported that female migrants may be at higher risk of cigarette [54, 55] and alcohol consumption [56] compared to males. However, since we did not have enough cases to stratify by sex, we could not explore this.

In addition, we have to highlight that the smoking status categorization we used in this study is primarily driven by frequency of consumption (having consumed cigarettes in the last month), without considering the amount component (how many cigarettes have been smoked). Thus, in our population, in which the number of cigarettes consumed is low, current smokers will have a lower smoking-related risk than current smokers in other countries with high smoking prevalence [57]. Also, our results suggest an overestimation of current smoking rates among rural dwellers when using definitions based on “occasionally/daily smoking”. Thus, studies using this definition may find different smoking rates and different measures of association than those found in our study.

## Conclusion

Our results show that migrants have a higher smoking prevalence than the rural population, but lower than the urban population, suggesting a potential protective effect of being exposed to a rural background. However, smoking incidence was similar in rural and migrant groups and higher in the urban group, suggesting that despite their current exposure to urban environments, migrants do not align with their urban peers. In addition, our results suggest that different definitions of smoking status could lead to different smoking rates and potentially different measures of association. On the other hand, heavy drinking prevalence and incidence were similar across all three population groups, suggesting similar heavy drinking culture and alcohol access between our urban and rural settings.

## Additional file

Additional file 1: Table S1. Outcomes of interest and evaluated co-variables. (DOCX 17 kb)

## Abbreviations

95% CI: 95% confidence intervals
GHQ-12: General Health Questionnaire
IQR: Interquartile ranges
PMH: Positive mental health
PR: Prevalence ratios
RR: Risk ratios
SD: Standard deviations
