# Supplementary material for: Smoking and heavy drinking patterns in rural, urban and rural-to-urban migrants: the PERU MIGRANT Study
Source: BMC Public Health. 2017 Feb 3;17:165. doi: 10.1186/s12889-017-4080-7 (PMC5291966; doi:10.1186/s12889-017-4080-7)
Supplement: Additional file 1: Table S1. — Outcomes of interest and evaluated co-variables. (DOCX 17 kb) [file 12889_2017_4080_MOESM1_ESM.docx]

## Supplementary table: Outcomes of interest and evaluated co-variables

| **Variables** | **Lifetime smoking N (%)** | | **Current smoking N (%)** | | **Heavy drinking N (%)** | |
| --- | --- | --- | --- | --- | --- | --- |
|  | **No** | **Yes** | **No** | **Yes** | **No** | **Yes** |
| Study group |  |  |  |  |  |  |
| Urban | 132 (67.3) | 64 (32.7) | 166 (84.7) | 30 (15.3) | 174 (91.1) | 17 (8.9) |
| Migrant | 492 (86.2) | 79 (13.8) | 534 (93.5) | 37 (6.5) | 522 (91.7) | 47 (8.3) |
| Rural | 175 (93.1) | 13 (6.9) | 180 (95.7) | 8 (4.3) | 174 (88.3) | 23 (11.7) |
| Sex |  |  |  |  |  |  |
| Female | 476 (95.2) | 24 (4.8) | 489 (97.8) | 11 (2.2) | 491 (97.6) | 12 (2.4) |
| Male | 323 (71.0) | 132 (29.0) | 391 (85.9) | 64 (14.1) | 379 (83.5) | 75 (16.5) |
| Age |  |  |  |  |  |  |
| < 50 years | 459 (84.7) | 83 (15.3) | 496 (91.5) | 46 (8.5) | 487 (88.9) | 61 (11.1) |
| ≥ 50 years | 340 (82.3) | 73 (17.7) | 384 (93.0) | 29 (7.0) | 383 (93.6) | 26 (6.4) |
| Education level |  |  |  |  |  |  |
| None or some primary education | 294 (93.6) | 20 (6.4) | 305 (97.1) | 9 (2.9) | 296 (93.1) | 22 (6.9) |
| Complete primary education | 128 (86.5) | 20 (13.5) | 136 (91.9) | 12 (8.1) | 135 (91.2) | 13 (8.8) |
| At least some secondary education | 376 (76.6) | 115 (23.4) | 438 (89.2) | 53 (10.8) | 437 (89.4) | 52 (10.6) |
| Assets index |  |  |  |  |  |  |
| Lowest | 371 (89.6) | 43 (10.4) | 393 (94.9) | 21 (5.1) | 388 (92.2) | 33 (7.8) |
| Middle | 187 (81.0) | 44 (19.0) | 212 (91.8) | 19 (8.2) | 214 (91.5) | 20 (8.5) |
| Highest | 241 (77.7) | 69 (22.3) | 275 (88.7) | 35 (11.3) | 268 (88.7) | 34 (11.3) |
| Positive mental health (mean ± SD) | 6.3 ± 1.8 | 6.8 ± 1.9 | 6.3 ± 1.8 | 7.0 ± 1.8 | 6.3 ± 1.8 | 6.8 ± 2.1 |
